# 90-gene signature assay for tissue origin diagnosis of brain metastases

**DOI:** 10.1186/s12967-019-2082-1

**Published:** 2019-10-01

**Authors:** Yulong Zheng, Yongfeng Ding, Qifeng Wang, Yifeng Sun, Xiaodong Teng, Qiqi Gao, Weixiang Zhong, Xiaofeng Lou, Cheng Xiao, Chengshu Chen, Qinghua Xu, Nong Xu

**Affiliations:** 1 Department of Medical Oncology, The First Affiliated Hospital, School of Medicine, Zhejiang University, Hangzhou, Zhejiang China; 2 Department of Pathology, Fudan University Shanghai Cancer Center, Shanghai, China; 3 Canhelp Genomics Co., Ltd., Hangzhou, Zhejiang China; 4 Department of Pathology, The First Affiliated Hospital, School of Medicine, Zhejiang University, Hangzhou, China; 5 Department of Surgical Oncology, The First Affiliated Hospital, School of Medicine, Zhejiang University, Hangzhou, China

**Keywords:** Primary brain tumor, Brain metastases, Gene expression profiling, Tumor origin identification, Quantitative real-time PCR

## Abstract

**Background:**

Brain metastases (BM) are the most common intracranial tumors. 2–14% of BM patients present with unknown primary site despite intensive evaluations. This study aims to evaluate the performance of a 90-gene expression signature in determining the primary sites for BM samples.

**Methods:**

The sequence-based gene expression profiles of 708 primary brain tumors (PBT) collected from The Cancer Genome Atlas (TCGA) database were analyzed by the 90-gene expression signature, with a similarity score for each of 21 common tumor types. We then used Optimal Binning algorithm to generate a threshold for separating PBT from BM. Eighteen PBT samples were analyzed to substantiate the reliability of the threshold. In addition, the performance of the 90-gene expression signature for molecular classification of metastatic brain tumors was validated in a cohort of 48 BM samples with the known origin. For each BM sample, the tumor type with the highest similarity score was considered tissue of origin. When a sample was diagnosed as PBT, but the similarity score below the threshold, the second prediction was considered as the primary site.

**Results:**

A threshold of the similarity score, 70, was identified to discriminate PBT from BM (PBT: > 70, BM: ≤ 70) with an accuracy of 99% (703/708, 95% CI 98–100%). The 90-gene expression signature was further validated with 18 PBT and 44 BM samples. The results of 18 PBT samples matched reference diagnosis with a concordance rate of 100%, and all similarity scores were above the threshold. Of 44 BM samples, the 90-gene expression signature accurately predicted primary sites in 89% (39/44, 95% CI 75–96%) of the cases.

**Conclusions:**

Our findings demonstrated the potential that the 90-gene expression signature could serve as a powerful tool for accurately identifying the primary sites of metastatic brain tumors.

## Background

Brain metastases (BM) are the most common neoplasms encountered in the central nervous system (CNS) and continue to be a major cause of mortality. It is estimated that between 9 and 17% of all newly diagnosed cancers will ultimately metastasize to the brain [1, 2]. The incidence is increasing with the development of improved imaging techniques and effective systemic treatment regimens, which prolong life. The frequency of brain metastasis is highest for lung cancer (40–50%), followed by breast cancer (15–25%) and melanoma (5–20%) [1, 3–5].

Traditionally, treatment options for BM, both known or unknown primary site, is limited and unsatisfactory, including surgical resection, whole brain radiotherapy (WBRT), radiosurgery, and chemotherapy [6]. Recently, based on accumulated data from a few retrospectives and small-sample prospective studies, researchers suggest that molecularly targeted systemic therapies may be an effective option for the treatment of BMs with the accurate known primary site, such as non-small-cell lung cancer [7, 8], breast cancer [9] and melanoma [10]. However, recent studies found that Vemurafenib, a selective inhibitor of BRAF^V600^, has shown significant response rate in BRAF^V600^ melanoma [11, 12], but not in metastatic BRAF^V600^ colorectal cancers [13], indicating the fundamentality of tumor tissue origin in molecular targeted therapy. Therefore, the accurate identification of the origin of BM is more important than ever for understanding the molecular underpinnings of tumors and facilitating patient-tailored therapy.

Generally, clinical symptoms, tumor markers, and imaging analysis help characterize the origin of metastatic neoplasms. However, these conventional approaches would get into the puzzle when the presumed primary tumor metastasizes before becoming large enough to be identified [14]. Specifically, 2–14% of BM patients present with no clearly detected primary site despite intensive evaluations [1, 3, 15]. In clinical practice, histopathology remains crucial for determining the anatomical origin and histological type of BMs. However, non-specific or inconclusive tissue morphology and immunohistochemical findings can confound, particularly when metastatic tumors are poorly differentiated or undifferentiated. Previous studies reported histopathological accuracies for diagnosing the primary site of BMs as low as 72.5% [16].

In recent years, gene expression profiling has become a useful tool for diagnostic [17], prognostic [18], predictive information for precise treatments selection [19], and for determining the origin of metastatic neoplasms [20]. Wu et al. applied a microarray-based 1550-gene expression profile to distinguish the tissue origin of BMs in 13 specimens of known origins and achieved good performance with an accuracy of 92.3% (12/13) [21]. In our preliminary study, we reported the identification of a 154-gene expression signature with an overall accuracy of 97% for the classification of 9626 carcinomas representing 22 tumor types [22]. Although the 22 tumor types cover the majority of tumor origins seen in adults, the primary clinical need is for identifying the origin of metastases, very often the lymph node metastases. It is uncommon for pathologists to be uncertain whether a tumor is a metastasis or a primary lymphoma; therefore, lymphoma was removed from the tumor panel. A modified version of the gene expression signature has been recently developed including 90 genes corresponding to 21 major tumor types [23]. Interestingly, but not surprisingly, Gene Ontology and KEGG pathway analysis of these 90 genes show that the most significant molecular features were “Pathways in cancer”, “Transcriptional misregulation in cancer”, “Prostate cancer”, “Pancreatic cancer” and so on.

In this study, we evaluated the utility of the 90-gene expression signature for molecular classification of metastatic brain tumors. Our results show that the 90-gene expression signature is a potentially useful diagnostic tool to identify the anatomical origin and histological type of BMs.

## Methods

### Sample selection

Study approval was obtained from the Ethics Committees of The First Affiliated Hospital, School of Medicine, Zhejiang University (Hangzhou, China) and Fudan University Shanghai Cancer Center (Shanghai, China). Between January 2012 and December 2017, primary brain tumors and brain metastases with known primary sites were entered in the study. In this study, the gold standard was the clinical features supplemented by morphology/immunohistochemistry (IHC) analysis. The primary tumor site was verified by clinical correlation of patient history and clinical, pathological and imaging information. Only tumor samples from 21 tumor types included in the 90-gene expression signature were selected (Additional file 1: Table S1, Additional file 2: Table S2). Formalin-fixed paraffin-embedded (FFPE) tissue samples were used for gene expression analysis. Before inclusion, hematoxylin and eosin (H&E)-stained slides from tumor samples were reviewed by two senior pathologists for sample quality control. Cases were excluded if tumor cells were fewer than 60% or necrotic area was more than 40%.

### RNA extraction

Total RNA was isolated from FFPE tumor tissue samples using an FFPE Total RNA Isolation Kit (Canhelp Genomics, Hangzhou, China) as described previously [24]. Briefly, the tumor tissue from 5 to 15 5-μm-thick paraffin sections was placed into 1.5 mL microcentrifuge tube, deparaffinized with xylene at 50 °C for 3 min and washed twice with 100% ethanol. Proteins were digested by incubation in a proteinase K solution at 56 °C for 15 min and then for another 15 min at 80 °C, following treatment with DNase. Total RNA was extracted using 40 μL RNase-free water. The concentration of total RNA was determined by spectrophotometer at 260 nm absorbance, and the purity of the extracted total RNA was determined by A260/A280 ratio. Gene expression analysis were only performed on RNA samples with A260/A280 ratios between 1.7 and 2.1.

### Gene expression profiling using quantitative real-time PCR

The 90 gene expression levels of brain tumor samples were measured by the quantitative real-time PCR (qRT-PCR) method as previously described [24]. For each specimen, cDNA synthesis performed on total RNA according to the protocol of High-Capacity cDNA Reverse Transcription Kit with RNase Inhibitor (Applied Biosystems, Foster City, CA, United States). Subsequently, the gene expression profiling was performed simultaneously on a 96-well plate using the Applied Biosystems 7500 Real-Time PCR (Applied Biosystems). The qRT-PCR cycling conditions were initiated at 95 °C for 10 min, followed by 40 cycles at 95 °C for 15 s and 60 °C for 1 min.

### Gene expression data analysis and similarity scores estimation

The 90-gene expression signature analyzed the expression pattern of each sample and generated similarity scores for each of 21 tumor types in the panel [22]. The similarity score measures how much is the gene expression pattern of the sample, similar to the global gene expression pattern of the indicated tumor type. Similarity score values ranged from 0 (low similarity) to 100 (high similarity) and summed up to 100 across all 21 tumor types in the panel. An example of a gene expression signature classification is shown in Additional file 3: Figure S1.

### Algorithm development and performance assessment

**Fig. 1:**
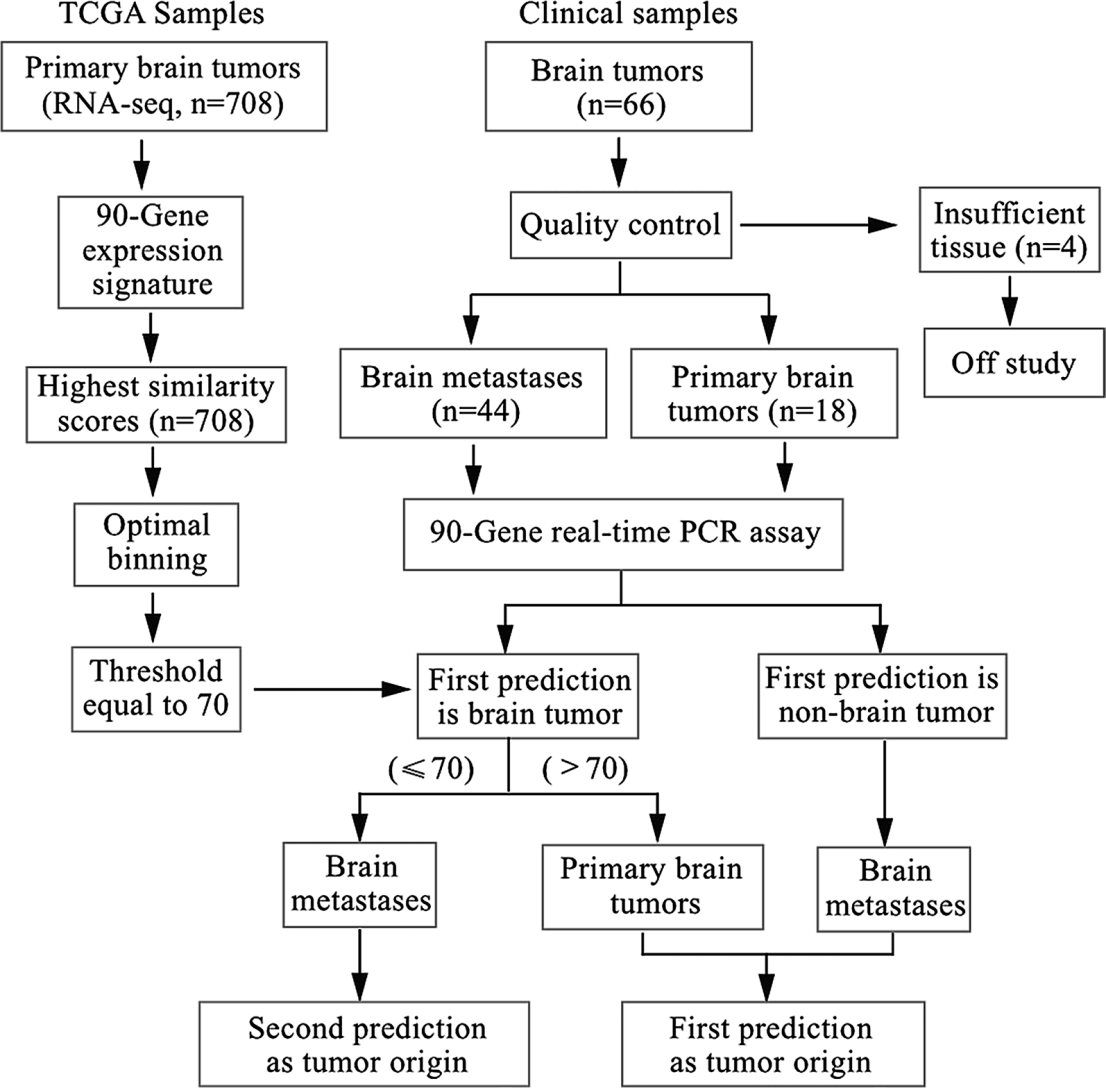
Case selection and flow diagram of the validation cohort through the study

Firstly, we calculated an optimal threshold to separate PBTs from BMs. The sequence-based gene expression profiling of 708 PBTs was collected from *The Cancer Genome Atlas* (TCGA) pan-cancer analysis working group at the Synapse website (https://www.synapse.org/). These data were generated from the Illumina HiSeq 2000 system consisting of transcriptomic data for 18,415 unique genes. The 90-gene expression signature was applied to the gene expression pattern of 708 samples. The highest similarity scores of 708 samples were analyzed using Optimal Binning algorithm in IBM SPSS software, and an optimum threshold was determined. Samples with the highest similarity score above the threshold were classified as PBTs, and those with the highest similarity scores below the threshold were considered as BMs (Fig. 1).

Secondly, we applied the 90-gene expression signature for each clinical specimen. The tumor type with the highest similarity score was considered as the tumor origin. However, for the cases with the highest similarity score below the threshold, but predicted as a brain tumor, the tumor type with the second highest similarity score was considered as the tumor origin.

For each clinical specimen, the predicted tumor type was compared with its reference diagnosis. The overall accuracy was defined as the number of correct cases divided by the total number of estimated cases. The hierarchical clustering of clinical specimens based on 90-gene expression profiles was performed using BRB-ArrayTools (version 4.5.1) [25].

## Results

### Patient characteristics

A total of 66 brain tumors with known primary were adopted from The First Affiliated Hospital, Zhejiang University, and Fudan University Shanghai Cancer Center in the study. Four metastatic brain samples were excluded due to insufficient tumor content. Sixty-two brain tumors met all quality control criteria and were analyzed by the 90-gene qRT-PCR assay. The demographics of 62 patients was characterized in Table 1. The cohort included 38 males and 24 females with a median age of 58.5 years, ranging from 6 to 84 years. The biopsy sites of all samples were the brain. Cases comprised 18 PBTs (29%) and 44 metastatic brain tumors (71%). The 18 PBTs comprise three subtypes that are meningiomas (n = 10), gliomas (n = 7) and primitive neuroectodermal tumor (n = 1). Based on the primary site of BMs, 44 samples were divided into six groups including lung (n = 26), colorectal (n = 6), breast (n = 6), neuroendocrine (n = 4), cervix (n = 1) and liver (n = 1). Among the 44 BM specimens, 18 (41%) cases were well-differentiated tumors and 26 (59%) cases were poorly differentiated tumors. For those poorly differentiated specimens, the morphology/IHC analysis correctly identified the primary sites in 18 of 26 (69.2%) BM cases.

**Table 1:** Patients information

Characteristic	No. of specimens (N = 62)	Percentage (%)
Age, years		
Median	58.5	
Range	6–84	
Gender		
Male	38	61
Female	24	39
PBTs		
Meningiomas	10	56
Gliomas	7	39
PNET	1	5
Origin of BMs		
Lung	26	59
Colorectal	6	14
Breast	6	14
Neuroendocrine	4	9
Cervix	1	2
Liver	1	2
Degree of differentiation in BMs		
Well-differentiated	18	41
Poorly differentiated	26	59

*BM* brain metastases, *PBT* primary brain tumor, *PNET* primitive neuroectodermal tumor

### Threshold identification for separating between PBTs and BMs

**Fig. 2:**
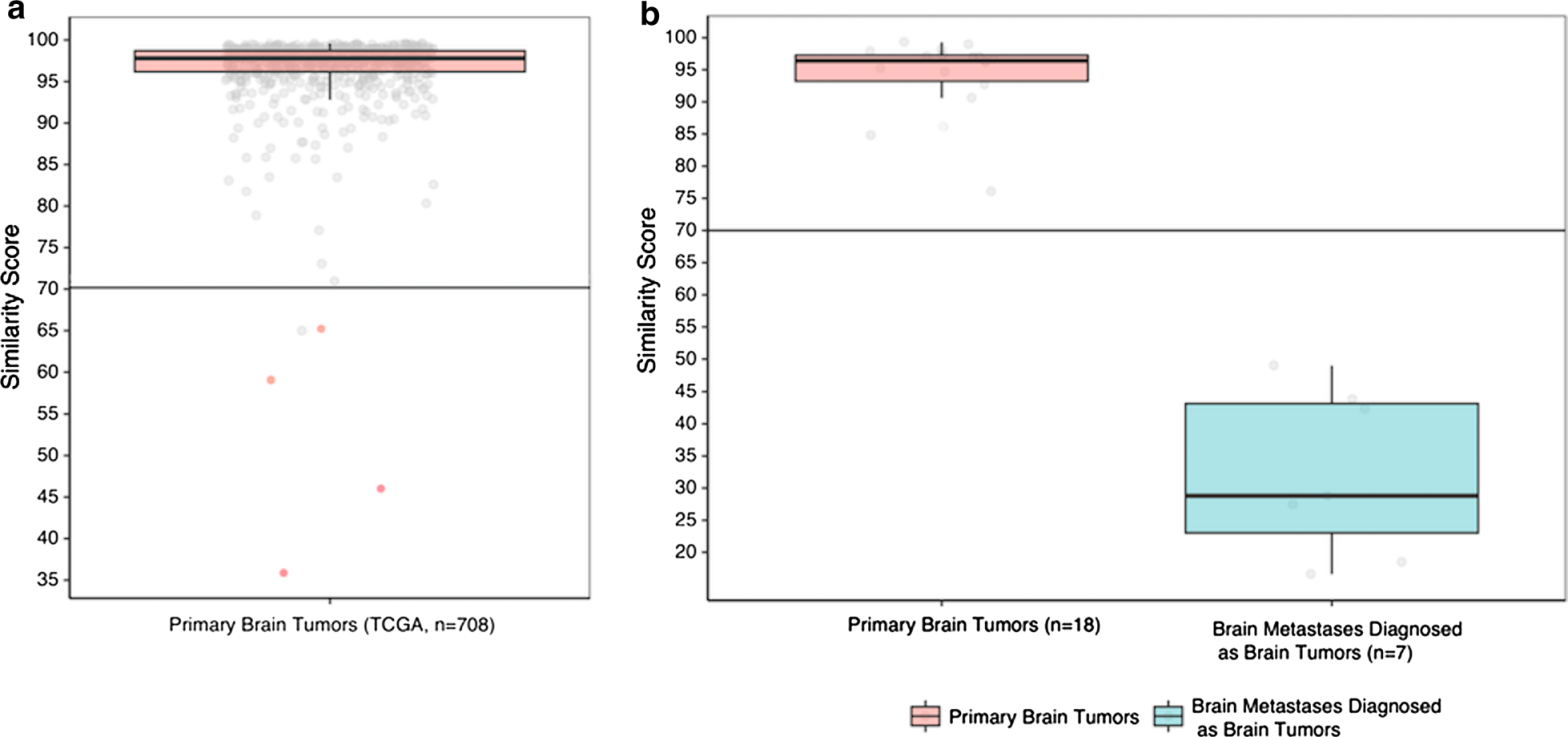
**a** Threshold identification for discrimination of PBTs and BMs. The grey spots indicate specimens corrected classified by 90-gene expression signature, whereas red spots show specimens with discordant results comparing with reference diagnosis. The similarity score of 70 (solid line) was identified as threshold by the Optimal Binning algorithm to discriminate PBTs and BMs. **b** Boxplot comparing the distribution of similarity scores of PBTs and BMs misclassified as brain tumors by 90-gene expression signature. The grey spots indicate the highest similarity scores of each specimens. The boxplots show the distribution of the highest similarity scores of PBTs (red) and misclassified BMs (cyan)

708 primary brain tumor samples achieved from TCGA were analyzed using the 90-gene expression signature. Through Optimal Binning algorithm analysis, a threshold of similarity score equal to 70 was established and was used to distinguish PBTs and BMs (Fig. 2a). Based on the threshold, the 90-gene expression signature predicted 703 of 708 samples with the highest similarity scores above 70 as PBT, and the remaining 5 samples considered as BM. Overall, the 90-gene expression signature showed a 99% agreement rate (703/708, 95% CI 98–100%) with reference diagnosis.

To substantiate the reliability of the threshold, an additional cohort of 18 PBTs and 44 BMs were analyzed by the 90-gene expression signature. The highest similarity score of 18 PBTs ranged from 76.1 to 99.3, with a median of 96.4. Among 44 BMs samples, 37 cases diagnosed as non-brain tumors had a median highest similarity score of 55.6, ranging from 22.2 to 97. Another 7 cases were predicted as PBTs, and the highest similarity scores ranged from 16.6 to 49, with a median of 28.8. The distribution of the highest similarity scores for 18 PBTs and 7 BMs misclassified as PBTs was shown in Fig. 2b. The highest similarity scores of PBTs were all above the threshold, whereas the highest similarity scores of misclassified BMs were all under the threshold. Therefore, seven BMs should further consider the second highest prediction as to the tumor of origin.

### Performance of the 90-gene expression signature in brain tumors

The performance of 90-gene expression signature in PBTs and BMs was shown in Table 2. The results of 18 PBT samples comprising meningiomas, gliomas, and primitive neuroectodermal tumor matched the reference diagnosis with an accuracy of 100%. Also, the 90-gene expression signature showed an 89% [39/44, 95% confidence interval (CI) 75–96%] agreement rate with the reference diagnosis in 44 BMs samples. For the 26 cases with the reference diagnosis of lung cancer, 21 samples were correctly classified with an accuracy of 81%. In addition, concordance rates were 100% for classifying the metastatic brain tumors from colorectal (n = 6), breast (n = 6), neuroendocrine (n = 4), cervix (n = 1) and liver (n = 1). For the poorly differentiated tumors, 21 out of 26 samples were correctly classified showing an accuracy of 81% (21/26, 95% CI 60–93%). Overall, 90-gene expression signature reached a 92% overall agreement with the reference diagnosis (57/62, 95% CI 81–97%).

**Table 2:** The performance of 90-gene expression signature in brain tumors

Reference diagnosis	No. of specimens	Agreement	Accuracy (%)
PBTs			
Meningiomas	10	10	100
Gliomas	7	7	100
PNET	1	1	100
Total	18	18	100
Origin of BMs			
Lung	26	21	81
Colorectal	6	6	100
Breast	6	6	100
Neuroendocrine	4	4	100
Cervix	1	1	100
Liver	1	1	100
Total	44	39	89

*BM* brain metastases, *PBT* primary brain tumor, *PNET* primitive neuroectodermal tumor

Five metastatic brain tumors had discordant predictions compared with reference diagnosis (Table 3). The histological types of five misclassified samples included lung squamous cell carcinoma (n = 4) and lung adenocarcinoma (n = 1). Two lung squamous cell carcinomas and one lung adenocarcinoma were predicted to be urinary carcinomas, one lung squamous cell carcinoma was adjudicated as a neuroendocrine tumor, and one lung squamous cell carcinoma was predicted to be a germ cell tumor.

**Table 3:** Investigation of cases with discordant 90-gene expression signature results

Case	Age	Gender	Resection	Grade	Immunohistochemistry	Reference diagnosis	Histology	Type prediction
1	53	Male	Cerebellum	Poorly differentiated	TTF-1(−), CK7(+), CK5/6(+), P63(+)	Lung	SCC	Neuroendocrine
2	75	Male	Cerebellum	Poorly differentiated	TTF-1(−), CK7(+), CK5/6(+), P63(−), CGA(−), CK(pan)(+), Ki-67(+), Syn(−)	Lung	SCC	Germ cell
3	57	Male	Cerebellum	Poorly differentiated	TTF-1(−), CK7(+), CK5/6(+), P63(+), CGA(−), CK(+), Syn(−), Napsin A(−), CDX2(−), CK20(−)	Lung	SCC	Urinary
4	62	Male	Cerebellum	Poorly differentiated	TTF-1(−), CK5/6(+), P63(−), GFAP(−), CK(pan)(+), Henatocyte(focal+), EBER(−), CDX2(−),	Lung	AC	Urinary
5	52	Male	Occipital lobe	Poorly differentiated	TTF-1(−), CK7(+), CK5/6(+), P63(+), GFAP(−), Vimentin(+), PR(−), EMA(focal+)	Lung	SCC	Urinary

*SCC* squamous cell carcinomas, *AC* adenocarcinomas

**Fig. 3:**
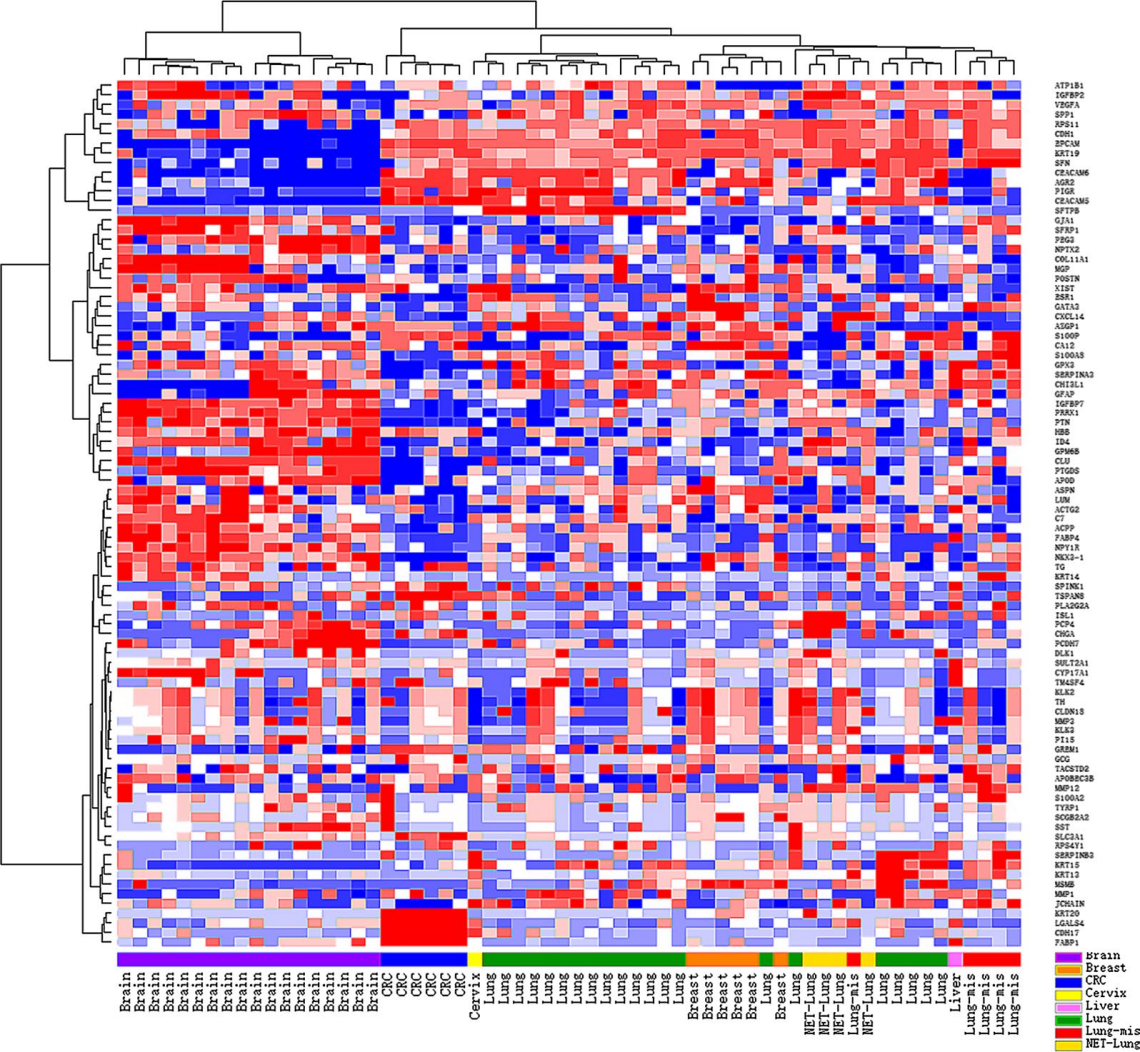
Hierarchical clustering analysis of 90 gene expression profiles in 62 brain tumor specimens. Normalized gene expression intensities were shifted to mean = 0, and rescaled to STD = 1 to enhance the expression differences. The average linkage hierarchical clustering method was performed where the metric of similarity was Pearson’s correlation between every pair of samples. The right panel indicates the official symbol of 90 genes and the left panel shows a dendrogram of hierarchical clustering of these genes. Colored pixels capture the magnitude of the expression for each gene, where shades of red and blue represent over-expression and under-expression, respectively, relative to the mean for each gene. The upper panel shows a dendrogram of hierarchical clustering of all samples. The bottom panel shows histological types including primary brain tumor (Brain, purple), breast cancer (Breast, orange), colorectal cancer (CRC, blue), cervix cancer (Cervix, yellow), liver cancer (Liver, pink), lung cancer (Lung, green), neuroendocrine tumor (NET-Lung, gold) and misclassified BMs (Lung-mis, red)

In order to evaluate the similarity between clinical samples, we performed hierarchical clustering. As shown in Fig. 3, the hierarchical clustering of 90 gene expression profiles in 62 samples revealed distinct patterns between six tumor types of BMs and PBTs. The breast cancer and neuroendocrine tumor samples were more similar to lung cancer.

## Discussion

BMs are the most common neoplasms encountered in the CNS and continue to be a significant cause of morbidity and mortality. The first step in the diagnosis of a metastatic brain lesion is to exclude a primary CNS tumor, followed by identification of tumor origin. In the clinic, the characteristics of brain tumor lesions (e.g., number, location) [26–28], advanced imaging techniques like PET-CT [29], and pathological exams may provide possible indications for distinguishing primary and metastatic brain tumors. However, when the metastatic brain tumor is poorly differentiated, morphology and IHC often fail to identify its anatomical origin and histological type [30]. Drlicek et al. proposed a combination of common immunohistochemical antibodies, for example, cytokeratin 7 (CK 7), thyroid transcription factor-1 (TTF-1), S100 protein and Carbohydrate antigen 19-9 (CA199), in the diagnosis of BMs with unknown primary. The combination approach was able to correctly identify the primary site in only 72.5% of BMs [16].

Several studies investigated the performance of genomic assays in identifying the primary site of BM. Alan et al. assessed the Tissue Of Origin (TOO) test that measures the expression pattern of 1550 genes to identify the primary site for BM patients. In a cohort of 13 cases, the test accurately classified 92.3% of patients [21], but the number of patients was too small to allow exploring true diagnostic performance. Although promising performance for the identification of tissue origin, the TOO test is unlikely for routine clinical use, due to its complexity and the cost of microarrays. Also, the TOO test did not include various squamous cell carcinoma in its test panel, which significantly narrows the value in determining the primary site of metastatic tumors, since squamous cell carcinoma represents a small but significant fraction of all cancer of unknown primary (CUP) cases. Mueller et al. described a microRNA-based test that classified 84% (75 of 89) of BMs using a qRT-PCR assay measuring 48 different microRNAs [31]. However, the algorithms of the microRNA-based test resulted in two possible tissues of origin, making it an inefficient diagnostic tool for physicians. Also, few data is supporting that both TOO test and microRNA-based test were capable of discriminating PBT from BM.

In the present study, we described the investigation of an effective and efficient approach for molecular classification of primary and metastatic brain tumors. By identification of the optimal threshold of similarity score equal 70, our 90-gene expression signature achieved an overall accuracy of 99% to classify PBT based on TCGA data. Additional validation of the threshold achieved an accuracy of 100% for classifying 18 PBTs and 44 BMs. Here, to our knowledge, this is the first report of a mRNA-based gene expression signature that can be used to discriminate primary and metastatic brain tumors. Even more interesting, the 90-gene expression signature achieved a precise classification of the primary tumor in 44 BM samples, with an overall accuracy of 89%. These results implied that the 90-gene expression signature might serve as a powerful tool for accurately identifying the tissue of origin for BM samples. Last but not least, the 90-gene expression signature could work with FFPE specimens, which allows widespread access and applications in clinical practice.

Although the 90-gene expression signature demonstrated highly accurate in classifying primary and secondary brain tumors, we noticed that five cases were misclassified. As shown in Table 3, the most obvious of these relates to poorly differentiated tumors. Given four of the five misclassified tumors are lung squamous cell carcinoma, it could be argued that squamous cell carcinomas are likely more susceptible to deterioration of gene expression pattern with increasing dedifferentiation. In subgroup analysis, the 90-gene expression signature achieved 80.8% of accuracy in total of 26 poorly differentiated BMs (21/26), which favorably compares with the 69.2% of accuracy by the traditional morphology/IHC analysis (18/26). In a blinded comparator study, Weiss et al. also demonstrated an overall accuracy of 79% by a 92-gene RT-PCR assay versus 69% by IHC/morphology analysis in the diagnosis of the primary site in metastatic tumors [32]. In line with these findings, our results suggest superior accuracy with the 90-gene expression signature versus standard-of-care morphology/IHC analysis and support the diagnostic utility of molecular classification in poorly differentiated BMs.

Traditional treatment options for BM, both known or unknown primary site, mainly focus on locoregional control of disease, which is with limited and unsatisfactory efficacy. Historically, the role of systemic therapy in the treatment of BM has also been limited [33]. In retrospective studies, the median overall survival for BM patients was less than 1 year [34, 35]. Recently, advances in several therapeutic modalities have effectively challenged the lethal status of brain metastasis for particular subsets of patients. Several targeted agents have shown improved systemic disease control and survival of selected BM patients [36], which have generated considerable interest in the investigation of these therapies to complement or even replace local therapies for the treatment of BM. Ceresoli et al. found that Gefitinib, an oral tyrosine kinase (TK) inhibitor of the epidermal growth factor receptor (EGFR), can be active on brain metastasis in non-small cell lung cancer (NSCLC) patients [7]. Lapatinib is an oral dual epidermal growth factor receptor and Her-2 inhibitor. Saleem et al. found that Lapatinib uptake was observed in brain metastasis of human epidermal growth factor receptor (HER)-2-positive breast cancer, but not in normal brain, suggesting that Lapatinib may have a role in the treatment of BM patients [9]. However, the efficacy of targeted agents varies widely based on the tumor type; e.g., the combination of BRAF and MEK inhibitors is known to be highly effective in melanoma patients with BRAF^V600^ mutations, while it has limited efficacy in colorectal cancer patients [37].

Given the immune checkpoint inhibitors have demonstrated significant and durable activity in a subset of patients with melanoma, lung cancer, bladder cancer, and many other malignancies, their activity has begun to be studied in patients with brain metastasis. A Phase 2 study showed that the PD-1 inhibitor pembrolizumab had activity in patients with untreated or progressive brain metastasis from melanoma or NSCLC [38]. Of 36 patients (18 with melanoma and 18 with NSCLC), brain metastasis response was achieved in 22% (4 of 18) patients with melanoma and 33% (6 of 18) patients with NSCLC. In a Phase 2 trail, CTLA-4 inhibitor ipilimumab demonstrated activity in brain metastasis in patients with melanoma [39]. 24% (12 of 51) patients who had stable and asymptomatic metastases achieved disease control with a median progression-free survival of 2.7 months and median overall survival of 7.0 months. Therefore, a precise diagnosis of tumor origin is more important than ever for the successful management of BM patients in the era of novel targeted therapies and immunomodulatory therapies.

## Conclusions

In conclusion, our findings demonstrated the potential that 90-gene expression signature might serve as a powerful tool for accurately identifying the tumor origin for BM patients. Future incorporation of the 90-gene expression signature in the BM diagnosis will assist oncologists in applying precise treatments, leading to improved care and outcomes for BM patients.

## Supplementary information

**Additional file 1: Table S1.** List of 21 tumor types.

**Additional file 2: Table S2.** List of 90 candidate genes.

**Additional file 3: Figure S1.** The gene expression profiling workflow. Total RNA from FFPE samples was extracted, followed by cDNA synthesis and qRT-PCR. Gene expression pattern was analyzed with 90-gene expression signature, with one similarity score for each of the 21 tumor types. The top 5 tissue with highest similarity scores in the sample report are as follows: Lung (70.7), Neuroendocrine (11.5), Germ cell (5.2), Liver (4.7), Colorectal (1.9), thus indicating the most likely tissue of origin is Lung (70.7).

## Data Availability

The dataset used and analyzed in the present study are available from the corresponding author on reasonable request.

## Abbreviations

BM: brain metastases
CA199: carbohydrate antigen 19-9
CK7: cytokeratin 7
CNS: central nervous system
CRC: colorectal cancers
EGFR: epidermal growth factor receptor
FFPE: formalin-fixed paraffin-embedded
H&E: hematoxylin and eosin
HER-2: human epidermal growth factor receptor-2
IHC: immunohistochemistry
NSCLC: non-small cell lung cancer
PBT: primary brain tumors
qRT-PCR: quantitative real-time PCR
TCGA: The Cancer Genome Atlas
TK: tyrosine kinase
TOO: tissue of origin
TTF-1: thyroid transcription factor-1
WBRT: whole brain radiotherapy
