# Supplementary material for: 90-gene signature assay for tissue origin diagnosis of brain metastases
Source: J Transl Med. 2019 Oct 1;17:331. doi: 10.1186/s12967-019-2082-1 (PMC6771090; doi:10.1186/s12967-019-2082-1)
Supplement: Supplementary file 1 — Additional file 1: Table S1. List of 21 tumor types. [file 12967_2019_2082_MOESM1_ESM.docx]

| **Table S1. List of 21 tumor types** |
| --- |
|  |
| Tumor types |
| Adrenal |
| Brain |
| Breast |
| Cervix |
| Colorectal |
| Endometrium |
| Gastroesophagus |
| Head and neck |
| Kidney |
| Liver |
| Lung |
| Melanoma |
| Mesothelioma |
| Neuroendocrine |
| Ovary |
| Pancreas |
| Prostate |
| Sarcoma |
| Testis |
| Thyroid |
| Urinary |
