# Supplementary material for: 90-gene signature assay for tissue origin diagnosis of brain metastases
Source: J Transl Med. 2019 Oct 1;17:331. doi: 10.1186/s12967-019-2082-1 (PMC6771090; doi:10.1186/s12967-019-2082-1)
Supplement: Supplementary file 2 — Additional file 2: Table S2. List of 90 candidate genes. [file 12967_2019_2082_MOESM2_ESM.docx]

| **Table S2. List of 90 Candidate Genes** | |
| --- | --- |
| **Gene Symbol** | **Description** |
| ACPP | acid phosphatase, prostate |
| ACTG2 | actin, gamma 2, smooth muscle, enteric |
| AGR2 | anterior gradient 2, protein disulphide isomerase family member |
| APOBEC3B | apolipoprotein B mRNA editing enzyme, catalytic polypeptide-like 3B |
| APOD | apolipoprotein D |
| ASPN | asporin |
| ATP1B1 | ATPase, Na+/K+ transporting, beta 1 polypeptide |
| AZGP1 | alpha-2-glycoprotein 1, zinc-binding |
| C7 | complement component 7 |
| CA12 | carbonic anhydrase XII |
| CDH1 | cadherin 1, type 1 |
| CDH17 | cadherin 17, LI cadherin (liver-intestine) |
| CEACAM5 | carcinoembryonic antigen-related cell adhesion molecule 5 |
| CEACAM6 | carcinoembryonic antigen-related cell adhesion molecule 6 (non-specific cross reacting antigen) |
| CHGA | chromogranin A |
| CHI3L1 | chitinase 3 like 1 |
| CLDN18 | claudin 18 |
| CLU | clusterin |
| COL11A1 | collagen, type XI, alpha 1 |
| CXCL14 | chemokine (C-X-C motif) ligand 14 |
| CYP17A1 | cytochrome P450 family 17 subfamily A member 1 |
| DLK1 | delta-like 1 homolog (Drosophila) |
| EPCAM | epithelial cell adhesion molecule |
| ESR1 | estrogen receptor 1 |
| FABP1 | fatty acid binding protein 1, liver |
| FABP4 | fatty acid binding protein 4, adipocyte |
| GATA3 | GATA binding protein 3 |
| GCG | glucagon |
| GFAP | glial fibrillary acidic protein |
| GJA1 | gap junction protein alpha 1 |
| GPM6B | glycoprotein M6B |
| GPX3 | glutathione peroxidase 3 |
| GREM1 | gremlin 1, DAN family BMP antagonist |
| HBB | hemoglobin subunit beta |
| ID4 | inhibitor of DNA binding 4, dominant negative helix-loop-helix protein |
| IGFBP2 | insulin like growth factor binding protein 2 |
| IGFBP7 | insulin like growth factor binding protein 7 |
| IGJ | Joining Chain Of Multimeric IgA And IgM |
| ISL1 | ISL LIM homeobox 1 |
| KLK2 | kallikrein related peptidase 2 |
| KLK3 | kallikrein related peptidase 3 |
| KRT13 | keratin 13, type I |
| KRT14 | keratin 14, type I |
| KRT15 | keratin 15, type I |
| KRT19 | keratin 19, type I |
| KRT20 | keratin 20, type I |
| LGALS4 | lectin, galactoside-binding, soluble, 4 |
| LUM | lumican |
| MGP | matrix Gla protein |
| MMP1 | matrix metallopeptidase 1 |
| MMP12 | matrix metallopeptidase 12 |
| MMP3 | matrix metallopeptidase 3 |
| MSMB | microseminoprotein, beta- |
| NKX3-1 | NK3 homeobox 1 |
| NPTX2 | neuronal pentraxin II |
| NPY1R | neuropeptide Y receptor Y1 |
| PCDH7 | protocadherin 7 |
| PCP4 | Purkinje cell protein 4 |
| PEG3 | paternally expressed 3 |
| PI15 | peptidase inhibitor 15 |
| PIGR | polymeric immunoglobulin receptor |
| PLA2G2A | phospholipase A2 group IIA |
| POSTN | periostin, osteoblast specific factor |
| PRRX1 | paired related homeobox 1 |
| PTGDS | prostaglandin D2 synthase |
| PTN | pleiotrophin |
| RPS11 | ribosomal protein S11 |
| RPS4Y1 | ribosomal protein S4, Y-linked 1 |
| S100A2 | S100 calcium binding protein A2 |
| S100A8 | S100 calcium binding protein A8 |
| S100P | S100 calcium binding protein P |
| SCGB2A2 | secretoglobin, family 2A, member 2 |
| SERPINA3 | serpin peptidase inhibitor, clade A (alpha-1 antiproteinase, antitrypsin), member 3 |
| SERPINB3 | serpin peptidase inhibitor, clade B (ovalbumin), member 3 |
| SFN | stratifin |
| SFRP1 | secreted frizzled-related protein 1 |
| SFTPB | surfactant protein B |
| SLC3A1 | solute carrier family 3 (amino acid transporter heavy chain), member 1 |
| SPINK1 | serine peptidase inhibitor, Kazal type 1 |
| SPP1 | secreted phosphoprotein 1 |
| SST | somatostatin |
| SULT2A1 | sulfotransferase family 2A member 1 |
| TACSTD2 | tumor-associated calcium signal transducer 2 |
| TG | thyroglobulin |
| TH | tyrosine hydroxylase |
| TM4SF4 | transmembrane 4 L six family member 4 |
| TSPAN8 | tetraspanin 8 |
| TYRP1 | tyrosinase-related protein 1 |
| VEGFA | vascular endothelial growth factor A |
| XIST | X inactive specific transcript (non-protein coding) |
