# Supplementary figures and images for: 90-gene signature assay for tissue origin diagnosis of brain metastases
Source: J Transl Med. 2019 Oct 1;17:331. doi: 10.1186/s12967-019-2082-1 (PMC6771090; doi:10.1186/s12967-019-2082-1)

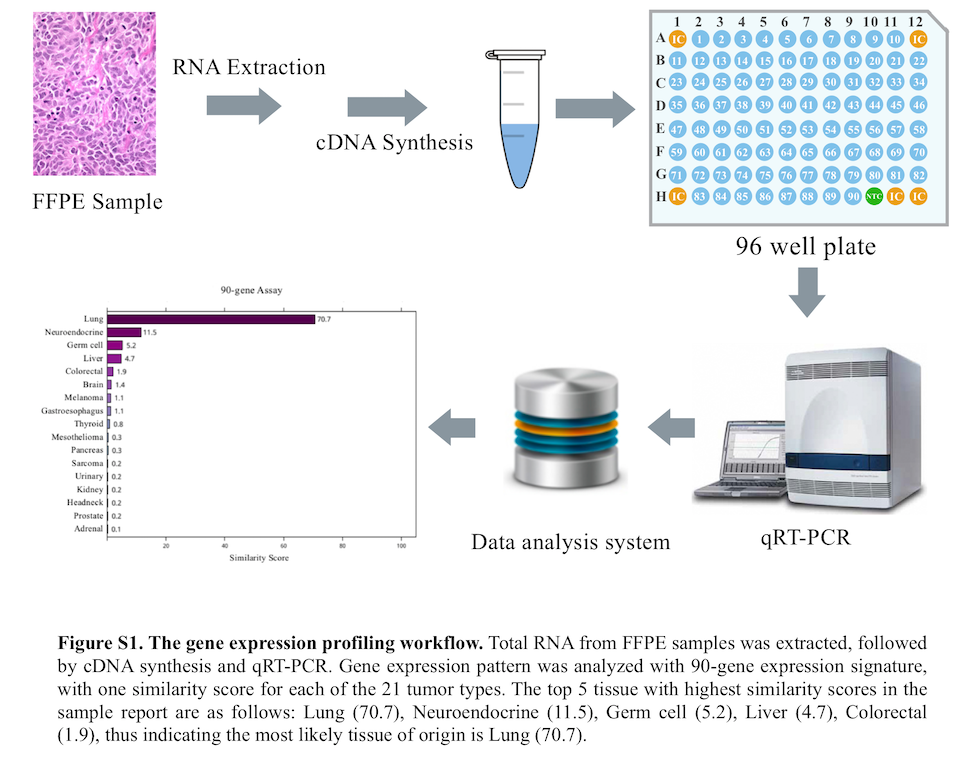

Supplement: Supplementary file 3 — Additional file 3: Figure S1. The gene expression profiling workflow. Total RNA from FFPE samples was extracted, followed by cDNA synthesis and qRT-PCR. Gene expression pattern was analyzed with 90-gene expression signature, with one similarity score for each of the 21 tumor types. The top 5 tissue with highest similarity scores in the sample report are as follows: Lung (70.7), Neuroendocrine (11.5), Germ cell (5.2), Liver (4.7), Colorectal (1.9), thus indicating the most likely tissue of origin is Lung (70.7). [file 12967_2019_2082_MOESM3_ESM.png]
